# Comparison of attitudes to breastfeeding among Spanish-born and Chinese-born postpartum women in Madrid

**DOI:** 10.1186/s13006-018-0187-4

**Published:** 2018-10-03

**Authors:** Juan Luis González-Pascual, Juana María Aguilar-Ortega, Laura Esteban-Gonzalo, Concepción Mesa-Leiva, Santiago Pérez-García, César Cardenete-Reyes

**Affiliations:** 1 School of Biomedical and Health Sciences, Nursing Department, Universidad Europea de Madrid (UEM), Villaviciosa de Odón, Madrid, Spain; 2 “12 de Octubre” Hospital, Madrid, Spain; 3 Universidad Rey Juan Carlos, Alcorcón, Madrid, Spain; 4 Universidad Complutense, Madrid, Spain

**Keywords:** Breastfeeding, Attitudes, Immigrant, Chinese, Spain, IIFAS

## Abstract

**Background:**

Maternal breastfeeding is a practice that is associated with multiple health benefits for mothers and children. One of the lowest rates of breastfeeding has been observed among Chinese women who immigrate to high income countries. At present, there is a lack of comparative information between this group and that of Spanish-born women. Considering the relationship between the attitude of women towards breastfeeding and the initiation of breastfeeding**,** the aim of the study was to determine whether the attitude towards breastfeeding among Chinese postpartum women who have immigrated to Spain differs from that of Spanish-born postpartum women.

**Methods:**

Cross-sectional study, with between-group comparison, of 73 postpartum Spanish-born and Chinese immigrant women admitted to the maternity units of “12 de Octubre” Hospital (Spain) between April and November 2016. Attitudes toward breastfeeding were analyzed using the Spanish or Chinese version of the Iowa Infant Feeding Attitude Scale (IIFAS). A wide set of socioeconomic, biological, working and attitudinal conditions were considered as covariates. The association between IIFAS and country of origin was assessed by three multiple linear regression models (B, SE, and 95% confidence interval were calculated).

**Results:**

All Chinese women were first generation immigrants. Chinese-born women were four years younger than Spanish-born mothers, had a lower educational level, more frequently had a paid job (mainly self-employed), and planned to return to work almost two months earlier than Spanish-born mothers did. Most Chinese women did not breastfeed exclusively.

Chinese immigrant women obtained scores of approximately 9 points less in the Iowa Infant Feeding Attitude Scale (IIFAS) when compared to Spanish-born women [95% CI -15.59, -2.48], after adjusting for the different socioeconomic, educational and work-related factors.

**Conclusions:**

Chinese-born women resident in Spain present a lower score on the IIFAS, when compared to Spanish-born women, which implies a more negative attitude towards breastfeeding. The between-groups difference is consistent, even when adjusting for known confounders and other factors which could affect the attitude of the mothers. It is therefore striking that, despite being in Spain, Chinese-born women maintain these preferences/attitudes regarding breastfeeding, compared with Spanish-born women, who obtain overall high scores.

## Background

It is well-known that maternal breastfeeding is a practice that is associated with multiple health benefits for mothers and children [1], both in the short [2], as well as the long term [3]. However, in high income countries, there are specific segments of the population who are less likely to follow this practice.

One such group is that of Chinese immigrant women. In this sense, in Canada, the rates of breastfeeding initiation are low among immigrant Chinese women [4, 5], between 8 and 48%, compared to 70% in the general population [5]. Also, in Australia, lower rates are recorded among Chinese immigrant women (75%) when compared with other groups (84%) [6]. According to other reports, rates in Chinese immigrant women may be similar comparing to other groups at first (74%), however decrease significantly after three months (50% compared to 63%) [7]. In Spain, where the migratory phenomenon is more recent, Chinese-born women are the group with lower rates of breastfeeding initiation (48%), compared to 80% of Spanish-born women and rates as high as 89% among immigrants in general (total mean rate of breastfeeding initiation) [8].

Several studies have shown that the decision to begin breastfeeding is associated with multiple factors: socio-occupational circumstances and those of the family setting [9], as well as certain intrinsic characteristics related to the mothers [10, 11]. These include the intention to breastfeed [12] and how the woman perceives breastfeeding [13]. Furthermore, other factors are constraints for breastfeeding initiation, specifically among the group of immigrant Chinese women. For example, cultural factors such as: the rest period after birth [14, 15]; the fact that the children are sent to China [15, 16]; work-related factors [15, 16]; or the influence of close friends and family [15, 17, 18].

Among these factors, the attitude of the mothers towards breastfeeding is key [12]. Few studies have been conducted on the attitude towards this practice among Chinese women who live in high income countries, and those that exist are inconclusive, ranging between less favorable [6], ambivalent [19], or favorable [20, 21].

One way of evaluating mothers’ attitudes is via the Iowa Infant Feeding Attitude Scale (IIFAS) [22]. This scale was originally developed and validated by de la Mora et al. [22] and has demonstrated to be useful for predicting the intention to breastfeed [23], exclusively breastfeeding upon hospital discharge [24, 25] and the duration of breastfeeding [26].

Thus, considering all the previously mentioned factor, we could conclude that attitudes regarding breastfeeding determine intention to breastfeed, which will contribute to the decision to begin breastfeeding and its initiation and duration.

Several factors have been identified that influence the scoring of the IIFAS. For example, the older the woman [22], and the better her socioeconomic status [21, 22] or educational level [21], the better her attitude towards breastfeeding is, in general. Conversely, if the woman intends to return to work [22], this has been associated with a more negative attitude towards breastfeeding.

## Methods

## Aim

Considering the low rates of breastfeeding among immigrant women from China, and our lack of knowledge regarding the attitude of these women towards breastfeeding (as a predictor of breastfeeding initiation), we devised this study to determine whether the attitude towards breastfeeding among Chinese postpartum women who have immigrated to Spain differs from that of Spanish-born postpartum women.

### Design

Cross-sectional study with between-group comparison.

### Participants

Study participants were recruited while being admitted to the post-natal maternity units of the 12 de Octubre Hospital, a metropolitan certified Baby-Friendly Hospital [27] in Madrid, Spain. This is the hospital to which most of the Chinese population in Madrid is referred (being the nearest hospital to the largest concentration of Chinese population in Spain) [28].

We estimated that a sample size of 30 participants was necessary in each group in order to detect differences of five or more points on the IIFAS score between groups, when considering a statistical power of 90%, a 95% confidence level and a variance of 36 points [29].

The study inclusion criteria were postpartum Spanish-born and Chinese-born women admitted at the maternity units of the hospital between April and November 2016. They were being treated within a period of 48–72 h after giving birth, during routine post-delivery health care. They had no diagnosis or significant complication. We excluded mothers with intensive care needs for either themselves or their child, or with mother/child diseases contraindicating breastfeeding, or those who had an out-of-hospital birth.

Consecutive sampling was carried out for Chinese women. To avoid a temporal bias, each day that a Chinese-born woman was interviewed, a Spanish-born woman was randomly selected among those admitted to the Maternity units, using a table with random numbers. A total of 42 Chinese women and 43 Spanish-born women were contacted, although 9 Chinese and 3 Spanish women declined to participate in the study. Thus, for the present analyses, valid data of 73 postpartum women aged 21–48 years was considered (33 Chinese-born and 40 Spanish-born women).

### Data collection

Data collection was carried out by nurses from the obstetrics units who were specially trained for collecting data, after written consent was obtained. The data were collected while the women were admitted to the hospital, the day that they were to be discharged (between 48 and 72 h after delivery). A self-reported questionnaire was used (except for the feeding method, which was determined by consulting data from the clinical history), available in Spanish and Chinese (an appropriate telephone translator was employed as required).

### Measures

#### The mothers’ attitude toward breastfeeding

This variable was analyzed using the Spanish or Chinese version of the IIFAS. This is a 17-item scale where respondents are asked to indicate the extent to which they agree with a series of statements based on a 5-point Likert scale (ranging from “strongly disagree” to “strongly agree”). Items 1, 2, 4, 6, 8, 10, 11, 14 and 17 are reverse-scored. The total score ranges between 17 and 85: the greater the score, the higher the preference is towards breastfeeding versus formula-feeding. The authors of the original version (English) considered the following interpretation of the score [22]:

- 81–85 Very Positive toward breast-feeding
- 70–80 Positive toward breast-feeding
- 49–69 Neutral
- 38–48 Positive toward formula-feeding
- 17–37 Very Positive toward formula-feeding

The IIFAS has been validated in different languages and countries, including both Spanish [30] and mainland Chinese versions [29].

#### Country of origin

The independent variable in this study was the immigrant status, determined by asking participants where they were born. Possible answers were Spain or China.

#### Length of residence in Spain

Only for Chinese-born women, the length of residence in Spain was self-reported in years, as an indirect measure of acculturation [31].

**Breastfeeding** was assessed by consulting the feeding method of each participant at discharge, as recorded in the clinical history for last feeding in hospital prior to discharge. Possible feeding methods were: exclusive breastfeeding, breastfeeding or formula-feeding [32]. For the present analyses, two categories were defined: exclusive breastfeeding and others.

#### Covariates

A wide set of socioeconomic, biological, working and attitudinal conditions were considered as covariates for the analyses, given their possible effect on IIFAS values: age (continuous variable), socioeconomic status (SES), level of education, paid work (yes/no), work status (self-employed/public administration employee/private employee), intention to return to work (yes/no), estimated time frame for returning to work and the partners’ attitude toward breastfeeding.

**Socioeconomic status** (SES)was assessed using the Family Affluence Scale (FAS), a four-item scale to measure material affluence, producing values that indicate low, medium or high SES [33].

**Mothers’ education level** was self-reported (primary school, secondary school, high school, university).

**Paid work** (work in exchange for a salary), **Return-to-work intention** and **Work status** were self-reported by the mothers.

**The estimated time frame for returning to work** was provided by the mothers based on self-reporting and measured in months as a continuous variable.

**The partner’s attitude toward breastfeeding** (positive, neutral and negative) was reported by the mothers.

**Intention to send the children** to be cared for by other family members outside the home was self-reported by the mothers.

### Analysis

Descriptive statistics are presented as the mean (standard deviation) or percentage (%). Statistical differences between Spanish-born and Chinese-born mothers were analyzed using the Student’s t-test or the chi-square test, as displayed in Table 1, in the results section. Statistical significance was determined by a two-sided *p* value (*p* < 0.05).

The association between IIFAS and the country of origin was assessed by multiple linear regression (B, SE, and 95% confidence interval were calculated). Three different models were built with the Spanish-born mothers as the reference group, with the objective of evaluating the potential influence of confounders, as well as to evaluate the influence of certain related variables on the relationship between the variables studied. In the first model, we adjusted for socioeconomic and biological confounders (SES, mothers’ educational level, age). In the second model, paid work and intention to return to work were included. In the third model, working conditions (return-to-work time frame and working status) and the partners’ attitudes toward breastfeeding were included. The analyses of interactions were carried out in several steps with no significant results. The goodness of fit was assessed using the Hosmer–Lemeshow test. Analyses were performed using the IBM SPSS Statistics 23.0 program for Windows (IBM, Armonk, New York).

## Results

Table 1 presents the characteristics of the sample (Chinese and Spanish-born women), as well as the results of the bivariate analysis. All Chinese women were first generation immigrants, whose average length of residence in Spain was 8.7 years (range 1 to 20 years). Women of Chinese origin were four years younger, had a lower educational level, more frequently had a paid job (mainly self-employed), and planned to return to work almost two months earlier compared to Spanish mothers (*p* < 0.05). Their partners’ opinion, referred by the Chinese mothers, was less supportive towards breastfeeding than that reported by the Spanish mothers (*p* < 0.05).

**Table 1:** Characteristics of the Spanish-born and the Chinese-born women in Madrid

Characteristic	Spanish women (*n*=40)	Chinese women (*n*=33)	*p* ^a^
Length of residence in Spain - years [mean, (SD)]	-	8.7 (4.1)	
Age [mean, (SD)]	33.1 (5.1)	28.7 (4.3)	<0.001
Socioeconomic status (SES) [n (%)]			0.115
High	11 (27.5)	6 (18.2)	
Medium	29 (72.5)	24 (72.7)	
Low	0 (0.0)	3 (9.1)	
Mother’s education level [n (%)]			<0.001
Primary school	1 (2.5)	5 (15.2)	
Secondary school	11 (27.5)	14 (42.4)	
High school	10 (25.0)	14 (42.4)	
University	18 (45.0)	0 (0.0)	
Partner’s positive attitude towards breastfeeding [n (%)]	38 (94.9)	25 (78.8)	0.030
Paid work (work in exchange for a salary) [n (%)]	29 (72.5)	32 (96.9)	0.017
Return-to-work intention [n (%)]	39 (97.5)	33 (100)	1.000
Work status [n (%)]			<0.001
Self-employee	4 (10.0)	21 (63.3)	
Public administration employee	10 (25.0)	1 (3.3)	
Employee	26 (65.0)	11 (33.3)	
Return-to-work time frame –months [mean, (SD)]	5.9 (2.7)	3.9 (1.8)	0.005
Intention to send the children to be cared for by other family members outside the home [n (%)]	0 (0.0)	4 (12.1)	0.025
Feeding at hospital discharge [n (%)]			<0.001
Exclusive Breastfeeding	32 (80.0)	12 (36.4)	
Others	8 (20.0)	21 (63.6)	

^a^Pearson’s x² test (Student’s t-test for age and return-to-work time frame)

The Chinese-born women showed lower adherence to exclusive breastfeeding (36.4%) compared with Spanish-born women (80%) (*p* < 0.05).

Table 2 shows the IIFAS scores in both groups as well as the results of the bivariate analysis. Women of Chinese origin presented a lower global score on the IIFAS test (59.3) compared with Spanish-born women (71.5) (*p* < 0.05).

**Table 2:** IIFAS scores of the Spanish-born and the Chinese-born women in Spain

Characteristic	Spanish women (*n*=40)	Chinese women (*n*=33)	*p* ^a^
IIFAS [mean, (SD)]			
Total score (17 - 85)	71.5 (6.9)	59.3 (7.3)	<0.001
Item 1^b^ score (1-5 all)	2.0 (1.3)	3.1 (1.3)	<0.001
Item 2^b^ score	1.3 (0.8)	2.9 (1.4)	<0.001
Item 3 score	4.7 (0.6)	4.6 (0.8)	0.577
Item 4^b^ score	2.1 (1.0)	2.6 (1.1)	0.054
Item 5 score	3.6 (1.2)	3.2 (1.1)	0.144
Item 6^b^ score	2.1 (1.0)	3.8 (1.2)	<0.001
Item 7 score	3.6 (1.2)	3.7 (1.0)	0.692
Item 8^b^ score	1.2 (0.5)	2.3 (1.1)	<0.001
Item 9 score	4.3 (1.0)	4.2 (1.1)	0.477
Item 10^b^ score	1.7 (0.8)	2.9 (1.0)	<0.001
Item 11^b^ score	1.6 (0.7)	2.2 (1.1)	0.006
Item 12 score	4.9 (0.4)	4.5 (0.9)	0.022
Item 13 score	4.5 (0.8)	4.5 (0.7)	0.809
Item 14^b^ score	1.8 (0.9)	2.9 (1.0)	<0.001
Item 15 score	4.9 (0.4)	4.2 (0.9)	<0.001
Item 16 score	4.8 (0.5)	3.8 (1.3)	<0.001
Item 17^b^ score	3.5 (1.3)	4.3 (1.0)	0.005

^a^ Student’s t-test

^b^Reverse-scored item

Table 3 displays the results of the three models of multiple linear regression regarding the association between the country of origin and the attitude towards breastfeeding.

**Table 3:** Multiple linear regression models for IIFAS score in Spanish-born and Chinese-born women in Spain

	Model 1^a^			Model 2^b^			Model 3^c^		
	B	SE	95% CI	B	SE	95% CI	B	SE	95% CI
Country of birth: China	-9.17	1.93	[-13.01, -5.32]	-9.62	2.48	[-14.60, -4.64]	-9.03	3.25	[-15.59, -2.48]

^a^ Model 1, adjusted for age, SES and mother’s education

^b^ Model 2, adjusted for age, SES, mother’s education, paid work and return-to-work intention

^c^ Model 3, adjusted for age, SES, mother’s education level, work status, return-to-work time frame and partner’s positive attitude toward breastfeeding

Being a Chinese-born woman was related with approximately 9 points less on the IIFAS survey when compared to Spanish mothers. The three models coincided in that these differences were maintained after adjusting both for known confounding variables, such as age, socioeconomic level and educational level, as well as considering work factors and the partner’s attitude towards breastfeeding.

## Discussion

In the present study, we found that Chinese-born women resident in Spain present a lower score on the IIFAS, when compared to Spanish-born women, which implies a more negative attitude towards breastfeeding. We did not find any change in the IIFAS score according to the length of residence in Spain, as an indirect measure of acculturation. The between-groups difference is consistent, even when adjusting for known confounders (age, socioeconomic status, educational level) [21, 22] and other factors which could affect the attitude of the mothers (work status, return-to-work time frame, partners’ attitude toward breastfeeding) [15, 17, 18, 22].

To the best of our knowledge, there are no previous studies comparing attitudes towards breastfeeding between Spanish and Chinese women to contrast our results, and studies comparing this issue between Chinese born-women and native women residing in other countries are scarce. In addition, most studies on the attitudes towards breastfeeding of Chinese-born mothers who are resident in high income countries do not use a validated scale such as the IIFAS, which makes comparisons with other studies difficult.

In line with our results, a study carried out in Australia compared early infant feeding decisions between English, Chinese and Arabic-speaking women during 1997 and 1998. Their conclusion was that Chinese-speaking women were less likely to express an intention to breastfeed and fewer initiated breastfeeding compared with other women [6]. Also, another study found that Chinese mothers living in Australia reported more positive attitudes towards breastfeeding when they were compared to Chinese mothers living in China [21].

In addition, another study exploring knowledge and attitudes related to breastfeeding of Chinese-born mothers residing in Ireland observed negative attitudes towards breastfeeding among those Chinese mothers examined [19]. In contrast, a qualitative Canadian study whose objective was to know the perceptions of Chinese mothers regarding breastfeeding found breastfeeding to be related to the value of common sense, purity of breast milk and the laws of nature in these women [20].

The global IIFAS score obtained by the present study in Chinese-born women was similar to that of Chinese-born women in Australia, 60 points [21]. However, this Australian study indicated that the results were positive as it compared these with women in China, whose score was slightly lower (57.7 points). This is despite the difference in the profile of the Chinese-born women in Spain and Australia. In our study, the socioeconomic and educational level of the mothers was medium or low, and most were self-employed. In Australia, the socioeconomic and educational level is high, and most do not work [21].

In our study, the mean IIFAS score in the sample of Chinese-born women was 59.3 points. In the study by Chen et al. [21], featuring women resident in China, the mean IIFAS score was 58 points, whereas, in another study, also based on IIFAS scores but focused solely on women of continental China, the score was 59 points [29], which is similar to our findings. According to the interpretation of the original version of the IIFAS score [22], scores obtained by Chinese-born women in the present study, as well as the scores obtained by Chinese-born women (both, residing in China and migrated to other countries) in the studies previously mentioned, showed neutral attitudes towards breastfeeding.

Other studies on women in China have highlighted that there is a lack of knowledge regarding the benefits of breastfeeding [34]. Furthermore, they believe that they have insufficient milk, whereas formula-feeding is considered as having multiple advantages [35, 36].

In our study, the mean IIFAS score in the sample of Spanish-born women was 71.5 points, which can be interpreted as positive attitudes towards breastfeeding [22]. Another study, also performed in Spain, reported a mean score of 66 points (mothers) [25]. These are high scores when compared with other high-income countries. Mothers from Canada score 64 points (pregnant women) [23], compared to 66 in Australia (mothers) [26], 61 in Japan (pregnant women) [24], 60 (pregnant) and 62 (mothers) in the United Kingdom [37], 58 (pregnant) [38] and 60 (mothers) in Scotland [39]. The scores obtained in all of them can be interpreted as neutral attitudes towards breastfeeding [22].

There is insufficient evidence to date to explain the causes underlying a less favorable attitude towards breastfeeding on behalf of Chinese-born women. However, it is interesting to note that the scores concerning breastfeeding among both Chinese women residing in China and immigrant Chinese-born women are very low compared to women living in high income countries. It is therefore striking that, despite being in Spain, Chinese women maintain these preferences/attitudes regarding breastfeeding, compared with Spanish women, who obtain overall high scores.

Socio-occupational factors, in general [9], and those related to the influence of significant people regarding the Chinese population, in particular [15, 17, 18], have demonstrated to be influential when initiating breastfeeding. However, in our study, none of these factors have shown to determine the attitude of Chinese-born women when compared to Spanish-born women regarding breastfeeding.

In our opinion, the data obtained in this study justify the performance of more extensive studies concerning the attitudes towards breastfeeding among immigrant Chinese women who have immigrated to high income countries. Data should be compared with that of country-born women and women in China, in order to determine the influence of both the associated cultural aspects (culture of the country of origin and influence of the acculturative process) and sociodemographic aspects (age, level of studies, type of work, etc.…).

This study is not without limitations. First, although the sample size may be sufficient for comparing the IIFAS score between groups, it is indeed modest, which may be associated with a limited statistical power for assessing some of the analyzed aspects. Second, the sample size and the fact that all women were recruited from the same hospital could hamper the generalization of these results. Third, part of the variables considered in this study are self-reported. Despite the validity of the tests used for these measurements, this could affect the quality of the data collected, which is a shortcoming inherent to the use of these questionnaires.

## Conclusion

Chinese-born women resident in Spain present a lower score on the IIFAS, when compared to Spanish-born women, which implies a more negative attitude towards breastfeeding. The between-groups difference is consistent, even when adjusting for known confounders (age, socioeconomic status, educational level) and other factors which could affect the attitude of the mothers (work status, return-to-work time frame, partners’ attitude toward breastfeeding). In our opinion, these results justify the necessity to design interventions to promote a more positive attitude towards breastfeeding among Chinese women.

This study contributes towards expanding the limited literature regarding the attitude towards breastfeeding of Chinese-born women residing in high income countries.

## Abbreviations

FAS: Family Affluence Scale
IIFAS: Iowa Infant Feeding Attitude Scale
SES: Socioeconomic Status

## References

[1] Victora Cesar G, Bahl Rajiv, Barros Aluísio J D, França Giovanny V A, Horton Susan, Krasevec Julia, Murch Simon, Sankar Mari Jeeva, Walker Neff, Rollins Nigel C (2016). Breastfeeding in the 21st century: epidemiology, mechanisms, and lifelong effect. The Lancet.

[2] Horta BL, Victora CG (2013). Short-term effects of breastfeeding: A systematic review on the benefits of breastfeeding on diarrhoea and pneumonia mortality.

[3] Horta BL, Victora CG. Long-term effects of breastfeeding: A systematic review. Geneva: World Health Organization; 2013.

[4] Chan-Yip A (2004). Health promotion and research in the Chinese community in Montreal: a model of culturally appropriate health care. Paediatric Child Health.

[5] Janssen PA, Livingstone VH, Chang B, Klein MC (2009). Development and evaluation of a Chinese-language newborn feeding hotline: a prospective cohort study. BMC Pregnancy Childbirth.

[6] Homer CSE, Sheehan A, Crooke M (2002). Initial infant feeding decisions and duration of breastfeeding in women from English, Arabic, and Chinese-speaking backgrounds in Australia. Breastfeed Rev.

[7] Li L, Zhang M, Scott JA (2005). Infant feeding practices in home countries and Australia: Perth Chinese mothers survey. Nutrition & Dietetics.

[8] Río I, Castelló-Pastor A, del Val Sandín-Vázquez M, Barona C, Jané M, Más R, Rebagliato M, Bolúmar F (2011). Breastfeeding initiation in immigrant and non-immigrant women in Spain. European Journal of Clinical Nutrition.

[9] Christopher GC, Krell JK (2014). Changing the breastfeeding conversation and our culture. Breastfeed Med.

[10] Wallwiener S, Müller M, Doster A, Plewniok K, Wallwiener CW, Fluhr H (2016). Predictors of impaired breastfeeding initiation and maintenance in diverse sample: what is important?. Arch Gynecol Obstet.

[11] Johnston ML, Esposito N (2007). Barriers and facilitators for breastfeeding among working women in the United States. J Obstet Gynecol Neonatal Nurs.

[12] Meedya S, Fahy K, Kable A (2010). Factors that positively influence breastfeeding duration to 6 months: a literature review. Women Birth.

[13] Díaz-Gómez M, Ruzafa-Martínez M, Ares S, Espiga I, De Alba C (2016). Motivations and perceived barriers to initiate or sustain breastfeeding among spanish women. Rev Esp Salud Publica.

[14] Lau Y, Htun TP, Lim PI, Ho-Lim SS, Chi C, Tsai C (2017). Breastfeeding attitude, health-related quality of life and maternal obesity among multi-ethnic pregnant women: a multi-group structural equation approach. Int J Nurs Stud.

[15] Lee Adele, Brann Lynn (2014). Influence of Cultural Beliefs on Infant Feeding, Postpartum and Childcare Practices among Chinese-American Mothers in New York City. Journal of Community Health.

[16] González-Pascual Juan L., Ruiz-López Montserrat, Saiz-Navarro Elena M., Moreno-Preciado Manuel (2015). Exploring Barriers to Breastfeeding Among Chinese Mothers Living in Madrid, Spain. Journal of Immigrant and Minority Health.

[17] Kuswara K, Laws R, Kremer P, Hesketh KD, Campbell KJ (2016). The infant feeding practices of Chinese immigrant mothers in Australia: a qualitative exploration. Appetite.

[18] Negin J, Coffman J, Vizintin P, Raynes-Greenow C (2016). The influence of grandmothers on breastfeeding rates: a systematic review. BMC Pregnancy Childbirth.

[19] Zhou Q, Younger KM, Kearney JM (2010). An exploration of the knowledge and attitudes towards breastfeeding among a sample of Chinese mothers in Ireland. BMC Public Health.

[20] Chen W (2010). Understanding the cultural context of Chinese mothers’ perceptions of breastfeeding and infant health in Canada. J Clin Nurs.

[21] Chen S, Binns CW, Liu Y, Maycock B, Zhao Y, Tang L (2013). Attitudes towards breastfeeding - the Iowa infant feeding attitude scale in Chinese mothers living in China and Australia. Asia Pac J Clin Nutr.

[22] de-la-Mora A, Russell DW, Dungy CI, Losch M, Dusdieker L (1999). The Iowa infant feeding attitude scale: analysis of reliability and validity. J Appl Soc Psychol.

[23] Twells LK, Midodzi WK, Ludlow V, Murphy-Goodridge J, Burrage L, Gill N (2016). Assessing infant feeding attitudes of expectant women in a provincial population in Canada: validation of the Iowa infant feeding attitude scale. J Hum Lact.

[24] Nanishi K, Jimba M (2014). Reliability and validity of the Japanese version of the Iowa infant feeding attitude scale: a longitudinal study. J Hum Lact.

[25] Tomás-Almarcha R, Oliver-Roig A, Richart-Martínez M (2016). Reability and validity of the reduced Spanish version of the Iowa infant feeding attitude scale. J Obstet Gynecol Neonatal Nurs.

[26] Cox KN, Giglia RC, Binns CW (2015). The influence of infant feeding attitudes on breastfeeding duration: evidence from a cohort study in rural Western Australia. Int Breastfeed J.

[27] Howe-Heyman A, Lutenbacher M (2016). The baby-friendly hospital initiative as an intervention to improve breastfeeding rates: a review of the literature. J Midwifery Womens Health.

[28] Estadística del padrón continuo [Census statistics]. Spanish Statistical Office, Spain. 2018. http://www.ine.es/dynt3/inebase/index.htm?type=pcaxis&path=/t20/e245/p04/provi&file=pcaxis&dh=0&capsel=2 . Accessed 19 Jul 2018.

[29] Hong-Xia D, Xiang-Dong G, Li X-M, You LM, Lau Y. Psychometric properties of a mainland Chinese version of the Iowa infant feeding attitude scale among postpartum women in China. Contemp Nurse. 2013;44(1):11–20. 10.5172/conu.2013.44.1.11 23721383

[30] Jácome Álvaro, Jiménez Ricardo (2014). Validación de la Iowa Infant Feeding Attitude Scale. Pediatría.

[31] Esteban-Gonzalo L, Veiga OL, Regidor E, Martínez D, Marcos A, Calle E (2015). Immigrant status, acculturation and risk of overweight and obesity in adolescents living in Madrid (Spain): the AFINOS study. J Immigr Minor Health.

[32] World Health Organization. Indicators for assessing infant and young child feeding practices: conclusions of a consensus meeting held 6–8 November 2007 in Washington D.C. USA. Geneva: World Health Organization; 2008.

[33] Currie C, Molcho M, Boyce W, Holstein B, Torsheim T, Richter M (2008). Researching health inequalities in adolescents: the development of the health behaviour in school-aged children (HBSC) family affluence scale. Soc Sci Med.

[34] Jiang H, Li M, Yang D, Wen LM, Hunter C, He G (2012). Awareness, intention, and needs regarding breastfeeding: findings from first-time mothers in Shanghai, China. Breastfeed Med.

[35] Tang L, Binns CW, Lee AH (2015). Infant formula crisis in China: a cohort study in Sichuan province. J Health Popul Nutr.

[36] Zhang K, Tang L, Wang H, Qiu L, Binns CW, Lee AH (2015). Why do mothers of young infants choose to formula feed in China? Perceptions of mothers and hospital staff. Int J Environ Res Public Health.

[37] Wilkins C, Ryan K, Green J, Thomas P (2012). Infant feeding attitudes of women in the United Kingdom during pregnancy and after birth. J Hum Lact.

[38] Dungy CI, McInnes RJ, Tappin DM, Wallis AB, Oprescu F (2008). Infant feeding attitudes and knowledge among socioeconomically disadvantaged women in Glasgow. Matern Child Health J.

[39] Scott JA, Shaker I, Reid M (2004). Parental attitudes toward breastfeeding: their association with feeding outcome at hospital discharge. Birth.

